# Precursors of Hypertensive Heart Phenotype Develop in Healthy Adults: A High-Resolution 3D MRI Study

**DOI:** 10.1016/j.jcmg.2015.08.007

**Published:** 2015-11

**Authors:** Antonio de Marvao, Timothy J.W. Dawes, Wenzhe Shi, Giuliana Durighel, Daniel Rueckert, Stuart A. Cook, Declan P. O’Regan

**Affiliations:** ∗ Medical Research Council Clinical Sciences Centre, Faculty of Medicine, Imperial College London, Hammersmith Hospital Campus, London, United Kingdom; † Department of Computing, Imperial College London, South Kensington Campus, London, United Kingdom; ‡ National Heart Centre Singapore, Singapore; § Duke–National University of Singapore Graduate Medical School, Singapore

**Keywords:** cardiac atlas, cardiac magnetic resonance, hypertension, left ventricular hypertrophy, remodeling, 2D, 2-dimensional, 3D, 3-dimensional, BP, blood pressure, CMR, cardiac magnetic resonance, FWT, fractional wall thickening, LV, left ventricular, RWT, relative wall thickness, SBP, systolic blood pressure, WS, wall stress, WT, wall thickness

## Abstract

**Objectives:**

This study used high-resolution 3-dimensional cardiac magnetic resonance to define the anatomical and functional left ventricular (LV) properties associated with increasing systolic blood pressure (SBP) in a drug-naïve cohort.

**Background:**

LV hypertrophy and remodeling occur in response to hemodynamic stress but little is known about how these phenotypic changes are initiated in the general population.

**Methods:**

In this study, 1,258 volunteers (54% women, mean age 40.6 ± 12.8 years) without self-reported cardiovascular disease underwent 3-dimensional cardiac magnetic resonance combined with computational modeling. The relationship between SBP and wall thickness (WT), relative WT, end-systolic wall stress (WS), and fractional wall thickening were analyzed using 3-dimensional regression models adjusted for body surface area, sex, race, age, and multiple testing. Significantly associated points in the LV model (p < 0.05) were identified and the relationship with SBP reported as mean β coefficients.

**Results:**

There was a continuous relationship between SBP and asymmetric concentric hypertrophic adaptation of the septum and anterior wall that was associated with normalization of wall stress. In the lateral wall an increase in wall stress with rising SBP was not balanced by a commensurate hypertrophic relationship. In normotensives, SBP was positively associated with WT (β = 0.09) and relative WT (β = 0.07) in the septal and anterior walls, and this regional hypertrophic relationship was progressively stronger among pre-hypertensives (β = 0.10) and hypertensives (β = 0.30).

**Conclusions:**

These findings show that the precursors of the hypertensive heart phenotype can be traced to healthy normotensive adults and that an independent and continuous relationship exists between adverse LV remodeling and SBP in a low-risk population. These adaptations show distinct regional variations with concentric hypertrophy of the septum and eccentric hypertrophy of the lateral wall, which challenge conventional classifications of LV remodeling.

At least one-quarter of the world’s adult population has hypertension and the increasing burden of disease has prompted efforts to search for earlier interventions and more effective therapeutic targets (1). Adverse structural and functional adaptations of the heart are of crucial relevance to the heightened cardiovascular risk associated with systemic hypertension, are frequently already established by the time therapy has been initiated 2, 3, 4, and are independent determinants of all-cause mortality 5, 6, 7. It remains uncertain what determines the onset and pattern of left ventricular (LV) remodeling, but the duration and severity of elevated blood pressure (BP), as well as genetic, metabolic, and environmental factors are all likely to be important (8). Although LV remodeling is known to begin at below-hypertensive levels, there is little data on how untreated healthy subjects adapt to rising BP, at what stage adverse LV changes are initiated and what mechanical factors drive hypertrophy 9, 10.

Our knowledge of the natural history of hypertensive heart disease in human populations has been developed from volume and mass measurements derived using conventional 2-dimensional (2D) echocardiography and cardiac magnetic resonance (CMR). Conventional classifications of hypertrophy and remodeling using LV mass/volume ratio rely on strong geometric assumptions about the anatomic uniformity of phenotypic adaptations (11). Three-dimensional (3D) CMR is a novel technique that has advantages over conventional phenotyping as it enables quantitative whole-heart assessment of cardiac physiology and noninvasive modeling of the predictors of LV morphology, function, and wall stress (WS) (12). In this study, we used 3D-CMR to define the relationship between systolic blood pressure (SBP) and region-specific LV adaptations in a population of healthy adults not taking antihypertensive medications.

## Methods

### Study population

In this study, 1,258 adult volunteers (680 women, age range 18 to 80 years, mean age 40.6 ± 12.8 years) were recruited prospectively via advertisement for the UK Digital Heart Project at Imperial College London. We excluded participants who had known cardiovascular or metabolic disease. Subjects taking prescription medicines were excluded but simple analgesics, antihistamines, and oral contraceptives were acceptable. Female subjects were excluded if they were pregnant or breastfeeding. Standard safety contraindications to magnetic resonance imaging were applied including a weight limit of 120 kg. All subjects provided written informed consent for participation in the study, which was approved by a research ethics committee.

### Participant phenotyping

#### Biophysical assessment

Each subject fasted for 4 h prior to the visit. Brachial BP measurement was performed following 5 min rest using a validated oscillometric device (Omron M7, Omron Corporation, Kyoto, Japan). The first of 3 measures was discarded and the second 2 values were averaged.

#### Cardiac magnetic resonance

CMR was performed on a 1.5-T Philips Achieva system (Best, the Netherlands). To capture the whole-heart phenotype, a high-spatial resolution 3D balanced steady-state free precession cine sequence was used that assessed the left and right ventricles in their entirety in a single breath-hold (60 sections, repetition time 3.0 ms, echo time 1.5 ms, flip angle 50°, field of view 320 × 320 × 112 mm, matrix 160 × 95, reconstructed voxel size 1.2 × 1.2 × 2 mm, 20 cardiac phases, temporal resolution 100 ms, typical breath-hold 20 s) (12). Conventional 2D cine imaging was also performed. Images were curated on an open-source image database (MRIdb, Imperial College London, United Kingdom) (13).

### Quantification of left ventricular mass and volume

Volumetric analysis of 2D LV cine images was performed using CMRtools (Cardiovascular Imaging Solutions, London, United Kingdom). Cardiac volumes and mass were indexed to body surface area.

### Three-dimensional assessment of ventricular structure and function

**Figure 1:**
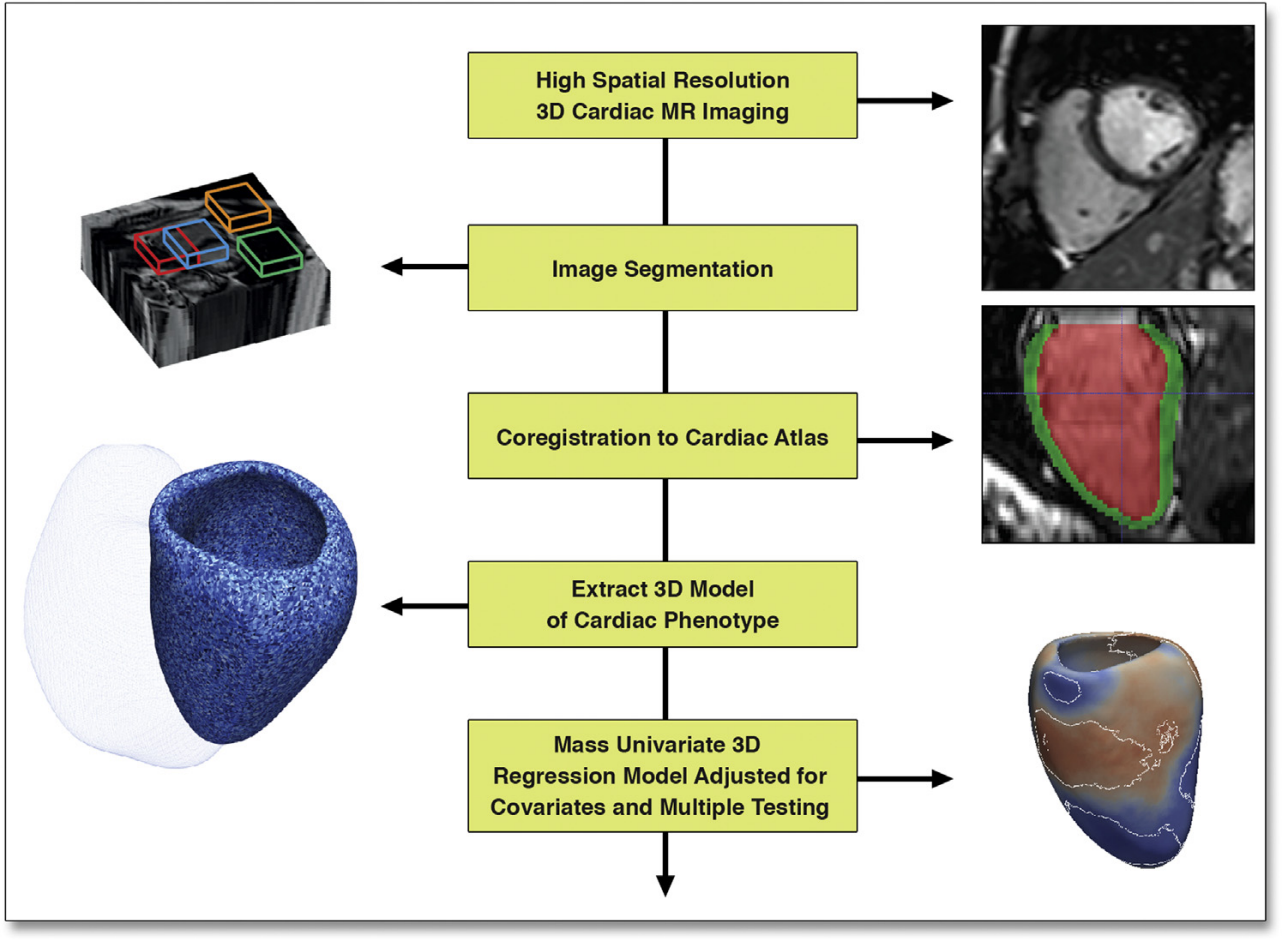
Statistical Model of How SBP Influences the LV Phenotype High spatial resolution imaging data is used to build a statistical model of how systolic blood pressure (SBP) influences the left ventricular (LV) phenotype. This paradigm allows the adaptations of whole heart structure and function in response to a stimulus to be explored. 3D = 3-dimensional.

Briefly, the heart was segmented from the 3D images using previous knowledge of cardiac anatomy from a set of manually annotated atlases (Figure 1) (14). Each segmentation was coregistered to ensure anatomical consistency between subjects. Wall thickness (WT) was calculated at over 40,000 points in the 3D model at end-diastole and was measured as the distance between the endocardial and epicardial surfaces perpendicular to the midwall plane. Relative wall thickness (RWT) was determined using a scale transformation of the myocardial surfaces to correct WT for variations in the size and shape of the LV. Differences in the endocardial and epicardial surfaces was determined relative to an average cardiac shape such that an outward or inward change would add or subtract from the volume on a regional basis. Fractional wall thickening (FWT) was calculated as: (end-diastolic WT − end-systolic WT)/end-diastolic WT × 100 (12).

The definitions of LV geometry used in conventional assessment of chamber size were adapted for 3D datasets (15). A regional hypertrophic response by the myocardium was defined as a positive regression coefficient between WT and SBP. A concentric pattern was present if there was a positive SBP correlation with RWT and a negative correlation with endocardial volume. Conversely, an eccentric pattern was indicated by a positive SBP correlation with endocardial volume and a negative correlation with RWT.

Regional end-systolic WS was determined at each point in the LV from the 3D Gaussian radius of curvature (R) and myocardial WT and calculated as follows (16):

$$Wall\;stress\propto SBP\;\times \;\frac{R}{WT}$$

### Statistical analysis

Data were analyzed using RStudio Server version 0.98 (Boston, Massachusetts) and Matlab (Mathworks, Natick, Massachusetts). Continuous variables are expressed as mean ± SD and categorical variables as percentages. The associations between phenotypic (WT, RWT, WS, and LV shape) parameters and SBP for each point in the 3D datasets were assessed using a regression model adjusted for age, sex, race, and body surface area with correction to control the false discovery rate (17). Contiguous regions of the LV where the association between variables was significant (p < 0.05) were identified and the relationship with SBP reported as the mean of the standardized β coefficients within that area. Comparison between groups was performed using analysis of variance, corrected for covariates, with effect size reported as η^2^ (18). When comparing 2 groups, Mann-Whitney *U* tests were used. Data from more than 2 groups were analyzed using a Kruskal-Wallis 1-way analysis of variance test followed by a Nemenyi post-hoc test for pairwise multiple comparisons.

## Results

### Study population characteristics

**Figure 2:**
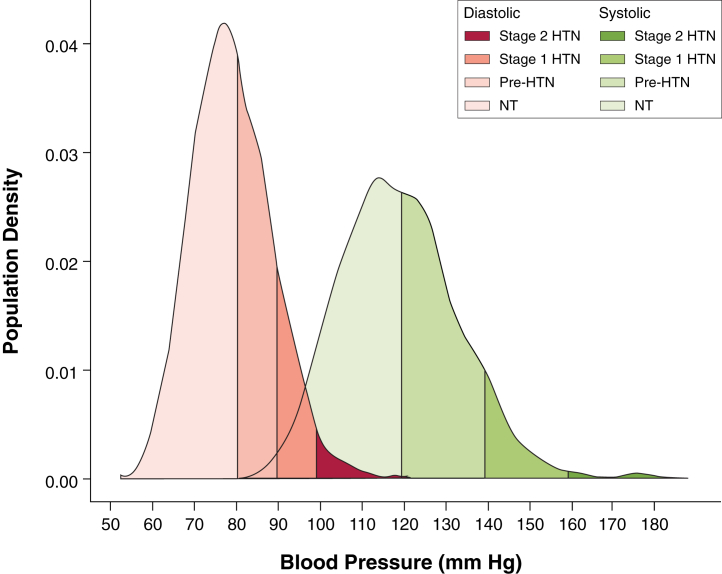
Density Plot Showing the Distribution of BP Readings in the Cohort The thresholds applied are those recommended in the Seventh Report of the Joint National Committee on Prevention, Detection, Evaluation, and Treatment of High Blood Pressure within our cohort (N = 1,258). BP = blood pressure; HTN = hypertension; NT = normotension.

According to the Seventh Report of the Joint National Committee on Prevention, Detection, Evaluation, and Treatment of High Blood Pressure criteria for SBP (19), of the 1,258 volunteers, 8.3% had stage 1 or 2 systolic hypertension (SBP ≥140 mm Hg), 38.4% had systolic pre-hypertension (SBP 120 to 139 mm Hg), and 53.3% had systolic normotension (SBP <120 mm Hg) as shown in Figure 2. A summary of subject characteristics split by these SBP thresholds is shown in Table 1. These data are presented for the whole cohort and separately for men and women in Online Table 1. A summary of the regression models using the conventional CMR data for the whole cohort is shown in Online Table 2. Among SBP subgroups, SBP was positively related to indexed LV mass in normotensives (β = 0.53, p < 0.001) and pre-hypertensives (β = 0.27, p < 0.05), but did not reach significance in hypertensives (β = 0.36, p = 0.06). Indexed LV mass and cardiac output were significantly lower in normotensives than in pre-hypertensives and hypertensives.

**Table 1:** Subject Characteristics and CMR-Derived Cardiac Measurements (N = 1,258)

	SBP Group			Effect Size (η^2^)	p Value		
	NT (n = 670 [53.3])	Pre-HTN (n = 483 [38.4])	HTN (n = 105 [8.3])		NT vs. Pre-HTN	NT vs. HTN	Pre-HTN vs. HTN
Sex					**<0.001**	**<0.001**	0.94
Male	213 (31.8)	298 (61.7)	67 (63.8)				
Female	457 (68.2)	185 (38.3)	38 (36.2)				
Age, yrs	37.6 ± 11.7	42.4 ± 13.0	51.0 ± 12.1		**<0.001**	**<0.001**	**<0.001**
Race/ethnicity							
Caucasian	483 (72.1)	378 (78.3)	89 (84.7)				
South Asian	91 (13.6)	54 (11.2)	10 (9.5)				
African	50 (7.5)	29 (6.0)	5 (4.7)				
Other	46 (6.8)	22 (4.6)	1 (1.0)				
Systolic BP, mm Hg	108.8 ± 7.1	127.7 ± 5.4	147.7 ± 9.1				
Diastolic BP, mm Hg	73.5 ± 6.5	83.2 ± 7.5	93.1 ± 8.9	0.33	**<0.001**	**<0.001**	**<0.001**
Height, cm	168.3 ± 8.9	171.9 ± 9.1	172.1 ± 10.7	0.0003	**<0.001**	**<0.001**	0.99
Weight, kg	67.8 ± 12.3	75.0 ± 12.4	77.5 ± 14.4	0.016	**<0.001**	**<0.001**	0.54
Body surface area, m^2^	1.77 ± 0.19	1.89 ± 0.19	1.92 ± 0.22	0.010	**<0.001**	**<0.001**	0.69
LVEDVI, ml/m^2^	79.0 ± 12.5	81.0 ± 14.3	79.7 ± 14.0	0.013	0.09	0.97	0.63
LVESVI, ml/m^2^	28.0 ± 7.3	28.1 ± 8.2	26.6 ± 7.8	0.003	0.97	0.14	0.19
LVSVI, ml/m^2^	51.0 ± 7.0	53.0 ± 8.0	53.0 ± 9.0	0.011	**0.001**	0.21	0.94
LVEF, %	64.9 ± 5.1	65.7 ± 5.6	66.9 ± 5.8	0.015	**0.03**	**<0.001**	0.05
LVMI, g/m^2^	59.5 ± 13.1	65.1 ± 14.4	69.0 ± 17.6	0.019	**<0.001**	**<0.001**	0.22
Cardiac output, l	5.8 ± 1.2	6.5 ± 1.5	6.6 ± 1.7	0.045	**<0.001**	**<0.001**	0.83
Heart rate, beats/min	63.9 ± 9.7	65.1 ± 10.8	65.8 ± 12.1	0.014			

Values are n (%) or mean ± SD. **Bold** values are statistically significant. Effect size of SBP group (NT vs. Pre-HTN vs. HTN) on each variable is presented after adjustment for age, race, and sex (η^2^ of ≥0.02 is a small effect, ≥0.13 is a medium effect, and ≥0.26 is a large effect). Kruskal-Wallis tests were carried out for each variable to determine whether differences between SBP groups were significant. When so, a post-hoc test was applied for pairwise multiple comparisons and those p values are shown.

BP = blood pressure; CMR = cardiac magnetic resonance; HTN = hypertension; LVEDVI = indexed left ventricular end-diastolic volume; LVEF = left ventricular ejection fraction; LVESVI = indexed left ventricular end-systolic volume; LVMI = indexed left ventricular mass; NT = normotension; SBP = systolic blood pressure.

### 3D LV geometry

**Figure 3:**
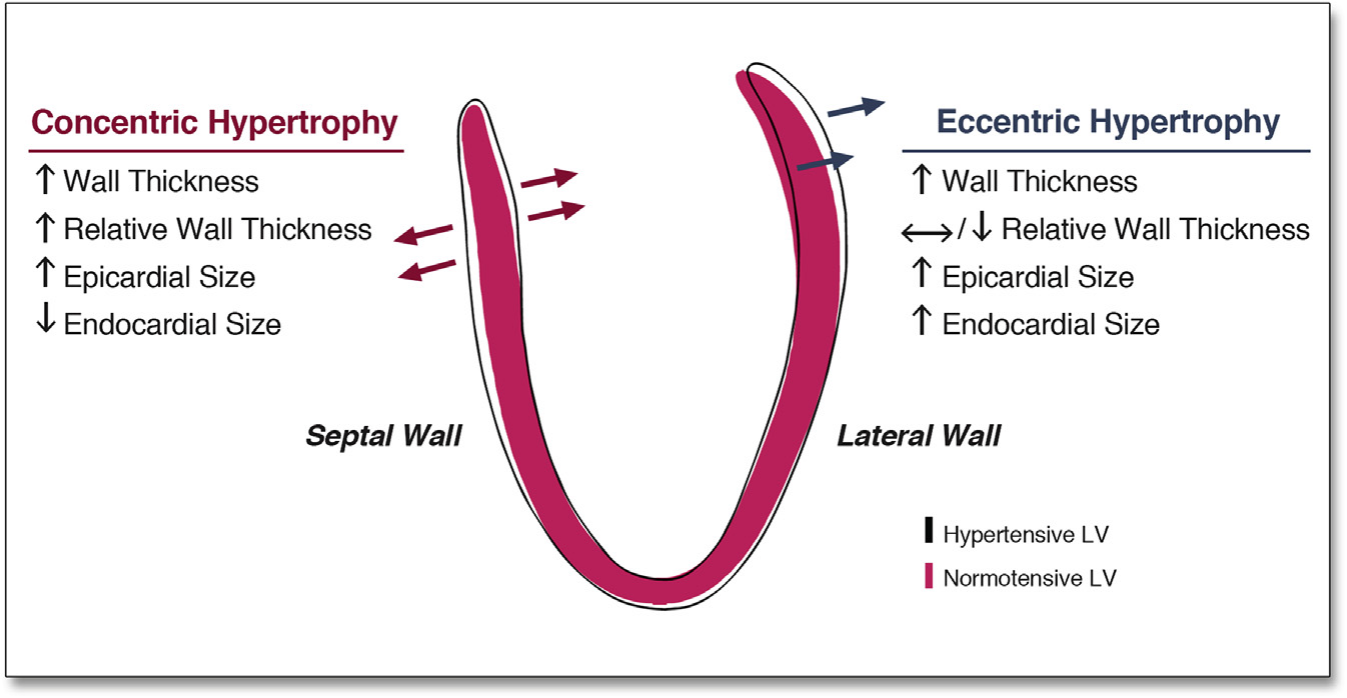
3D Model of the Regional Changes in LV Geometry Associated With SBP A long-axis section of the 3D cardiac magnetic resonance–derived fitted regression model taken at SBP of 100 mm Hg **(red filled contour)** and 180 mm Hg **(black outline)** shows how LV geometry varies between these 2 BP. **Arrows** indicate the relationship between each coefficient and SBP. Abbreviations as in Figure 1.

The pattern of LV hypertrophy and remodeling observed with respect to each of the phenotypic variables is illustrated in Figure 3 (and in each sex in Online Figure 1).

In normotensives, the 3D regression models revealed a positive relationship between SBP and WT throughout the LV (β = 0.08, significant area = 99%) with the strongest effect observed in the septum and anterior wall. Controlling for ventricular volume, a positive relationship was also found between SBP and RWT that was strongest in the mid-ventricular anterior and septal walls (β = 0.07, significant area = 25%) but was not observed in basal or lateral walls (Figure 4, Online Video 1).**Figure 4:**
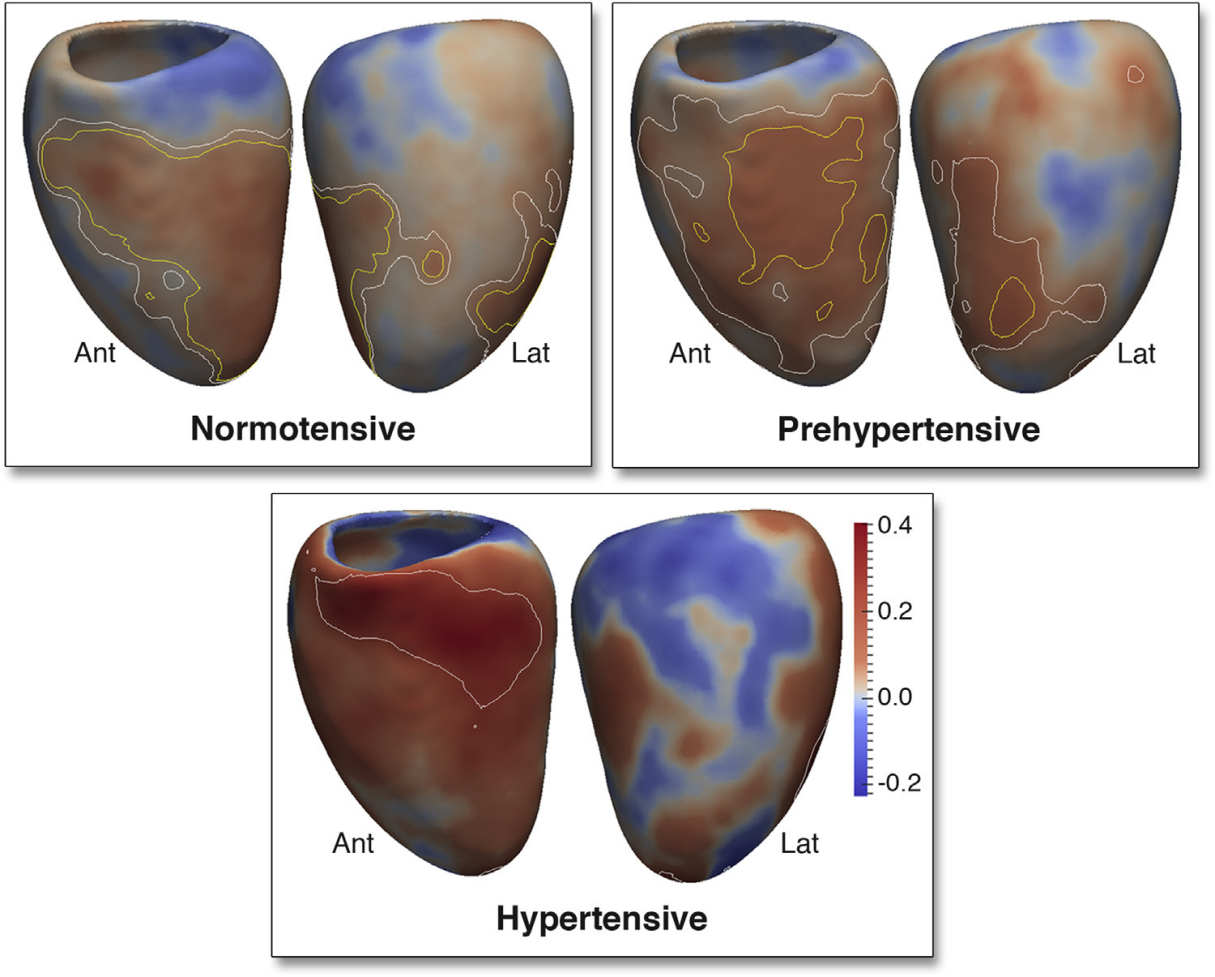
3D Regression Models of the Association Between SBP and LV RWT in NT, Pre-HTN, and HTN Adults The regression coefficients between SBP and LV relative wall thickness (RWT) are shown for subjects categorized by the thresholds of the Seventh Report of the Joint National Committee on Prevention, Detection, Evaluation, and Treatment of High Blood Pressure. Positive coefficients indicate concentric hypertrophy and negative coefficients eccentric hypertrophy. **Contour lines** indicate significant regions (p < 0.05) before **(white border)** and after **(yellow border)** correction for multiple testing, respectively. LV projections are anterior (Ant) and lateral (Lat). Please see Online Video 1. Abbreviations as in Figures 1 and 2.

**Figure:**
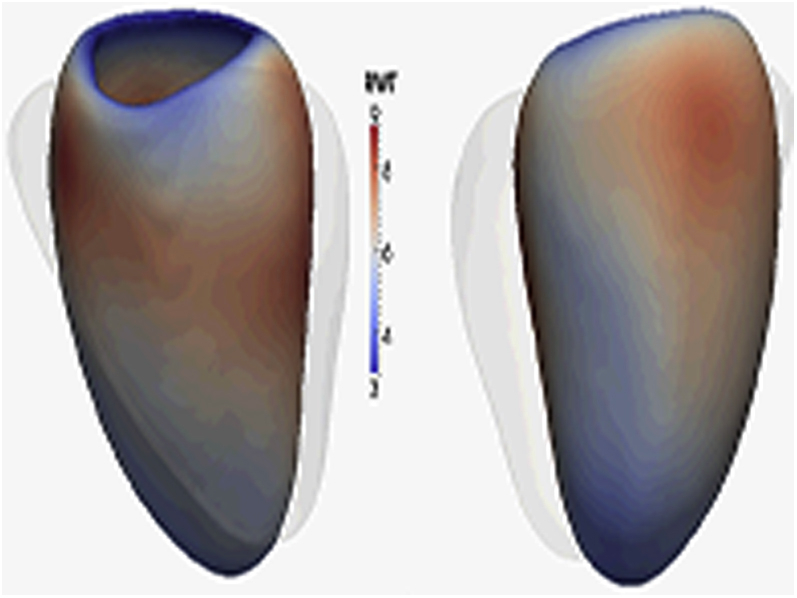
Online Video 1

In pre-hypertensives, WT was positively related to SBP throughout the septal and anterior walls including the apico-anterior and lateral walls (β = 0.11, significant area = 15%). The greatest hypertrophic response in RWT was observed in the mid-ventricular anterior and septal walls (β = 0.10, significant area = 8%) with negative coefficients elsewhere.

In hypertensives, there was a strong and asymmetric association between SBP and both WT (β = 0.42, significant area = 0.5%) and RWT (β = 0.30, significant area = 9.6%) that was observed in the basal septal and anterior walls, with negative coefficients in the lateral wall.

**Figure 5:**
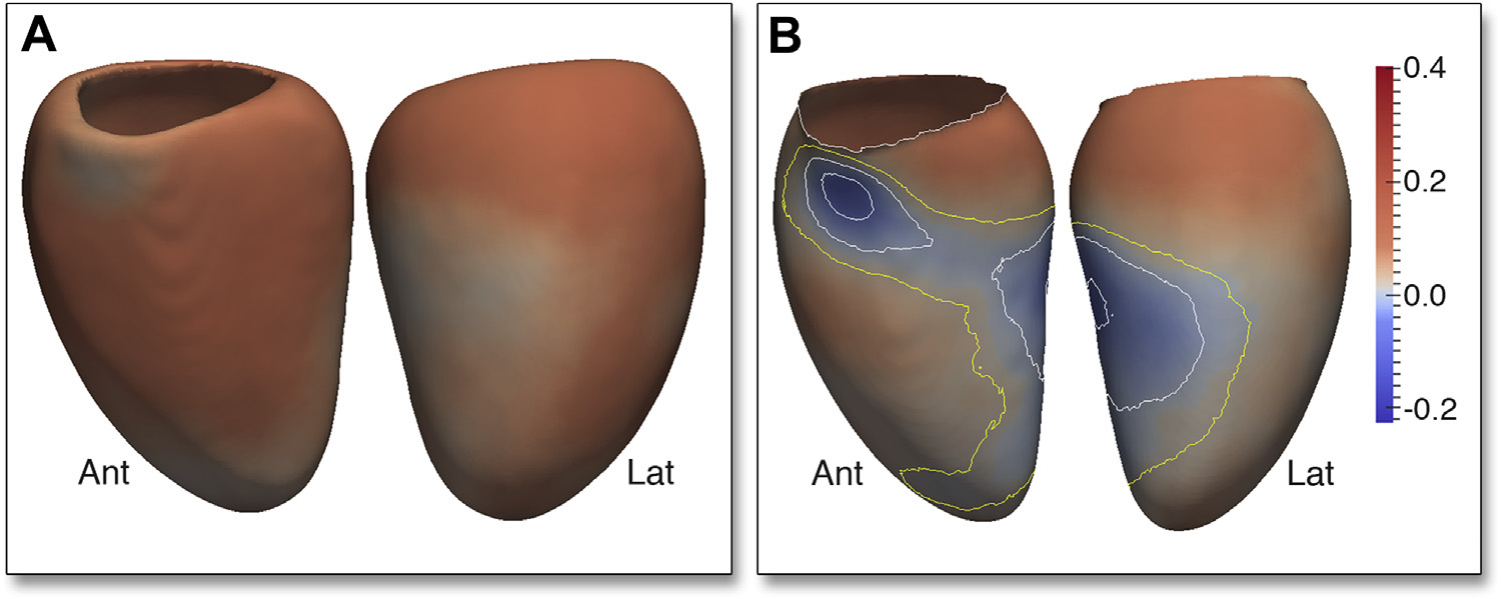
3D Regression Models of the Association Between SBP and LV Geometry Across the Cohort The regression coefficients between SBP and LV shape are shown for the epicardial **(A)** and endocardial **(B)** surfaces. A positive coefficient indicates an outward expansion of the surface and a negative coefficient an inward contraction. **Contour lines** indicate significant regions (p < 0.05) before **(white border)** and after **(yellow border)** correction for multiple testing. Whereas the majority of the LV exhibits eccentric hypertrophy, the septum and anterior wall undergo concentric hypertrophy. Abbreviations as in Figures 1 and 4.

Overall, there was an outward increase in the LV epicardial surface, relative to the mean shape, associated with SBP (β = 0.08, significant area = 97%). The majority of the LV endocardial surface also showed an outward expansion in response to SBP (β = 0.06, significant area = 51%). Taken together, these data show a predominantly eccentric hypertrophic pattern in response to SBP. However, in a region from the basal anteroseptal wall to the mid-ventricular anterolateral wall, the increase in WT was associated with an inward shape change of the endocardial surface (β = −0.02, significant area = 4.6%) at the expense of the LV cavity (Figure 5). Considering the changes in both RWT and endocardial volume, increasing SBP is characterized by a pattern of regional concentric hypertrophy in the septum and anterolateral wall and eccentric hypertrophy elsewhere.

### 3D LV function

In normotensives, FWT was positively associated with SBP in the anterior and septal walls and lateral wall (β = 0.10, significant area = 25.2%), indicating preserved or hyperdynamic radial function across SBP in normotension. In pre-hypertensives, the positive relationship between SBP and FWT was limited to the mid-ventricular lateral and inferior walls (β = 0.09, significant area = 15.7%) with negative coefficients in the basal septum and anterior walls. In hypertensives, FWT had stronger negative association with SBP, which was localized to the basal and mid-ventricular septum (β = −0.23, significant area = 3.2%), revealing reduced radial function colocalized to the area of greatest hypertrophic response.

### 3D wall stress

**Figure 6:**
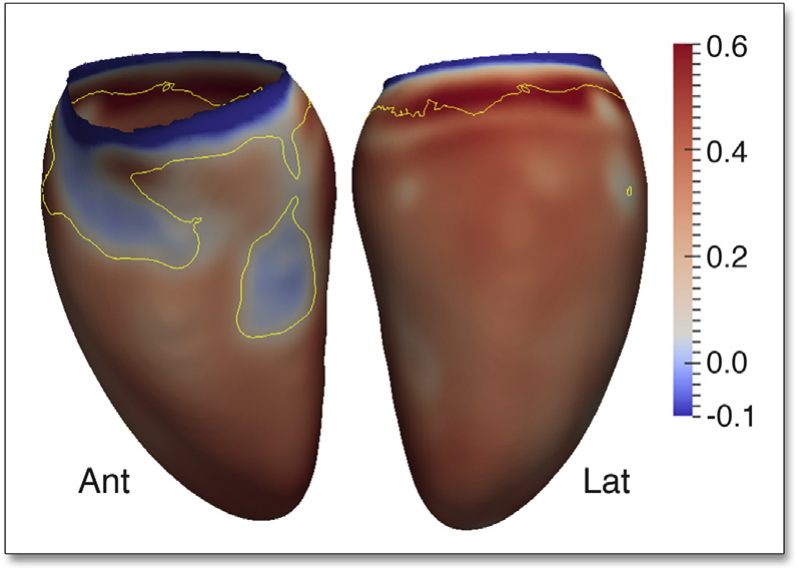
3D Regression Models of the Association Between SBP and Regional End-Systolic WS Across the Cohort The regression coefficients are shown in the endocardial surface with positive coefficients indicating increased wall stress (WS) with rising SBP. **Yellow contour lines** indicate significant regions (p < 0.05) after correction for multiple testing. In areas of septal concentric hypertrophy, the increase in WS is matched by an increase in WT; however, elsewhere there is a significant increase in WS in response to rising SBP. LV projections are Ant and Lat. Abbreviations as in Figures 1 and 4.

Across the full cohort, WS was strongly positively associated with SBP (β = 0.35, significant area = 81.1%) in the majority of LV, demonstrating an uncompensated increase in WS. However, in the basal anteroseptum and mid-ventricular anterior wall, there was no significant association between WS and SBP. This demonstrates that only in these regions was the increase in BP-dependent WS compensated for by proportionate concentric hypertrophy (Figure 6).

## Discussion

This study provides new insights into the effects of SBP on LV morphology and function using advanced cardiovascular phenotyping and computational modeling. Accurate representations of cardiac morphology can be obtained using atlas-based methods, and this approach provides new opportunities for understanding the regulation of LV hypertrophy in humans and can be applied to large population-level datasets for mechanistic or interventional studies 12, 20, 21. Our findings show that the precursors of the hypertensive heart phenotype can be traced to healthy normotensive adults and that an independent and continuous relationship exists between adverse LV remodeling and SBP in a low-risk population.

A central dogma of experimental studies is that pressure overload causes elevated WS, which stimulates a concentric pattern of compensatory hypertrophy 22, 23. However, such responses are not purely compensatory as load-induced hypertrophy does not always have adaptive value (24) and an augmented contractile state can occur without hypertrophy (25). Both LV mass (3) and SBP (26) show a continuous and independent relationship with disease risk, but the mechanisms underlying these associations remain elusive. In contrast to conventional classification systems, which make strong assumptions about the uniformity of hypertrophy and remodeling, we have demonstrated that there is a strong and distinct regionality to the homeostatic response of myocardium in the face of rising SBP in which concentric and eccentric adaptations occur concurrently 11, 27, 28, 29.

We observed that rising SBP is associated with a normalization of Laplacian WS in the septum where concentric hypertrophy is predominant. However, in the majority of the LV, the increase in WS with rising SBP was not balanced by a proportionate increase in RWT. High WS without signs of decompensation has been reported in uncomplicated hypertension due to an increase myocardial contractility in the early stages of disease 30, 31 with regional geometric differences in remodeling that are most pronounced in the basal septum (32). In keeping with this, we observed preserved or hyperdynamic radial function in normotensives with rising SBP, despite early geometric changes. However, in hypertensives, this relationship was reversed and function was most diminished in the regions exhibiting the greatest hypertrophy. The mechanisms underlying this anatomic asymmetry of hypertensive hypertrophy and remodeling is only partly understood but may reflect the spiral trajectory of subepicardial fiber architecture, the influence of titin isoform expression on myocardial compliance, embryonic origins of myocardial regions, and local variations in both mechanoreceptors and mechanical loading 33, 34.

Prognostically adverse cardiovascular features, including LV hypertrophy and increased vascular stiffness, have been observed in pre-hypertensives with a high prevalence of obesity and diabetes (35). Our data show that a “hypertensive pattern” of remodeling is also observed in asymptomatic normotensive adults. This suggests that rising SBP may have a much earlier impact on cardiac structure and function than previously recognized and might point toward a nuanced relationship between homeostasis and cardiovascular risk. It was notable that with rising SBP the increase in septal RWT is progressively more pronounced, and this has been previously recognized as a phenotype of hypertension especially in the elderly (36). The mechanism for increasing hypertrophic sensitivity to rising SBP remains to be determined and may depend on vascular, mechanical, and/or neurohormonal factors that contribute to the acceleration in the regional RWT increase as SBP rises 37, 38, 39.

### Study limitations

A cross-sectional study cannot determine the causal relationships between increased SBP and LV geometry. We categorized subjects according to the thresholds in the Seventh Report of the Joint National Committee on Prevention, Detection, Evaluation, and Treatment of High Blood Pressure, as more recent guidelines have not addressed the definitions of hypertension or pre-hypertension (40). We performed BP measurement according to European Society of Hypertension guidelines (41), but ambulatory monitoring is considered the reference standard for confirming elevated office readings (42). BP readings were therefore not simultaneous with the CMR. We used a simple model of Laplacian WS in our regression model to demonstrate the potential relationship between afterload and hypertrophy. Our 3D datasets provide an accurate patient-specific model of curvature and WT that does not rely on geometric assumptions; however, we assumed that material properties of the myocardium were uniform as we did not have data on mechanical characteristics or fiber orientations in this cohort. Therefore, the relationship between WS and hypertrophy is subject to unmeasured factors and should be interpreted cautiously. Although 3D imaging offers advantages for whole-heart phenotyping, the images also have lower signal-to-noise ratio and poorer temporal resolution compared with those seen with 2D imaging (12). Effect sizes in healthy individuals were more modest compared with those in the hypertensive range of SBP. We did not assess subjects for insulin resistance or subclinical diabetes, which is thought to influence the pattern of LV remodeling, although findings have been inconsistent (43), nor did we account for the potential effects of smoking or alcohol consumption. We controlled for racial group, but this study did not have sufficient power to explore ethnic differences in LV remodeling (44). Each vertex in the 3D model was considered independently of its neighbors and cluster-based significance thresholding could be more sensitive for detecting extended areas of anatomical association.

## Conclusions

The precursors of the hypertensive heart phenotype can be traced to healthy normotensive adults and a continuous relationship exists between adverse LV remodeling and SBP in a low-risk population. Rising SBP is associated with concentric hypertrophy of the septum and eccentric hypertrophy of the lateral wall. These findings challenge the conventional understanding of compensated cardiac hypertrophy in pressure overload.

Perspectives **COMPETENCY IN MEDICAL KNOWLEDGE:** The precursors of the hypertensive cardiac phenotype are apparent in healthy normotensive adults. In contrast to current classifications of remodeling, the LV makes strong and distinct regional adaptations to rising SBP, which encompass both concentric and eccentric patterns of hypertrophy.

**TRANSLATIONAL OUTLOOK:** High-resolution 3D imaging with computational analysis provides an automated and objective means to assess the environmental and genetic determinants of whole-heart structure and function. Future epidemiological research should apply these methods in prospective studies to determine how cumulative exposure to elevated SBP leads to hypertrophy in susceptible individuals.
