# Supplementary material for: Precursors of Hypertensive Heart Phenotype Develop in Healthy Adults: A High-Resolution 3D MRI Study
Source: JACC Cardiovasc Imaging. 2015 Nov;8(11):1260–9. doi: 10.1016/j.jcmg.2015.08.007 (PMC4639392; doi:10.1016/j.jcmg.2015.08.007)
Supplement: Online Figure 1 and Online Tables 1 and 2 [file mmc1.docx]

**Supplementary Material**

**Online Figure 1:**

**Three dimensional regression models of the association between systolic blood pressure and left ventricular relative wall thickness in normotensives, pre-hypertensives and hypertensives by gender.**

The regression coefficients between systolic blood pressure and left ventricular relative wall thickness are shown for subjects categorized by JNC-7 thresholds in males and females. Positive coefficients indicate concentric hypertrophy and negative coefficients eccentric hypertrophy. Left ventricular projections: Ant = Anterior; Lat = Lateral.


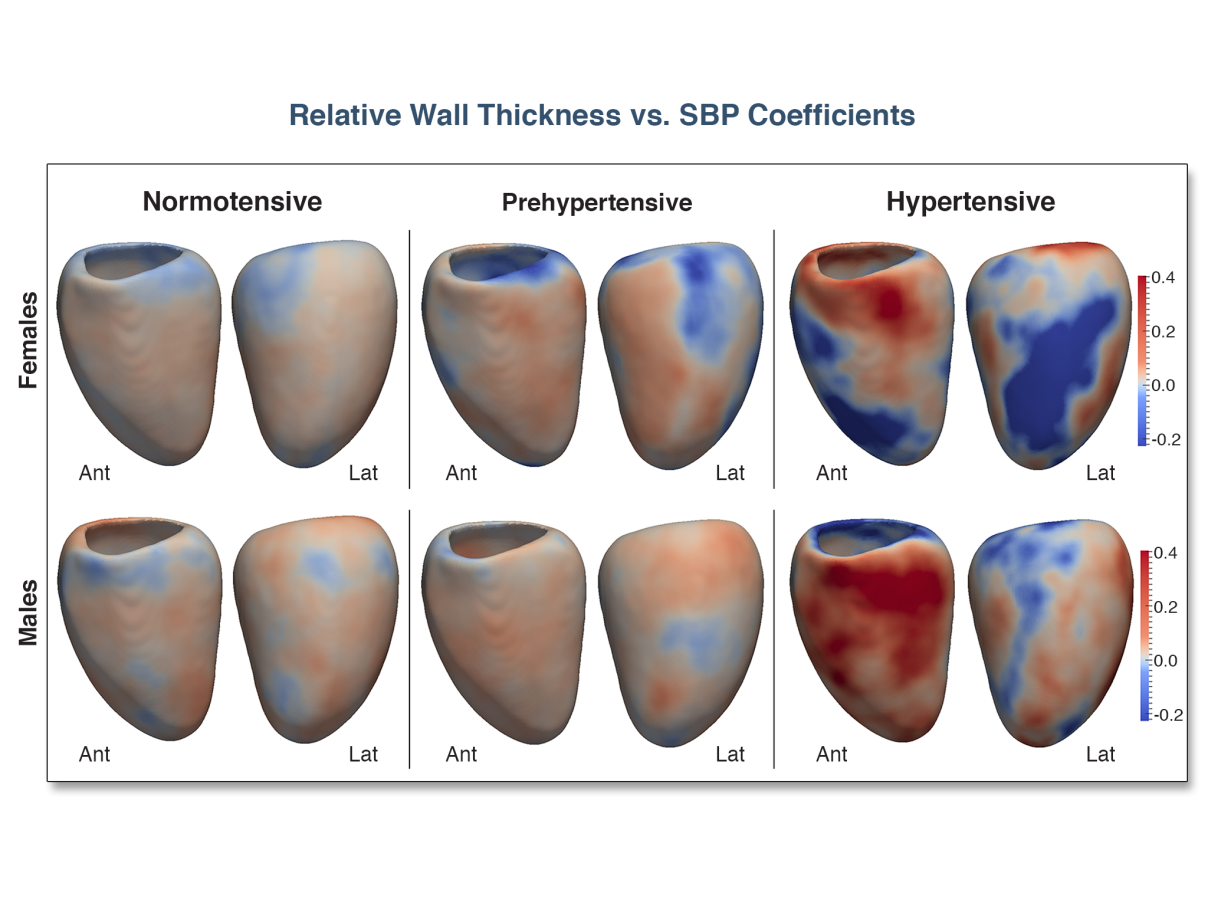


**Online Table 1:** Subject characteristics and CMR-derived cardiac measurements for the whole cohort (n = 1258) and split by gender. Values are in mean ± SD with ranges or percentages in brackets. P-values for pairwise comparisons derived from Mann Whitney U tests. BP = blood pressure; BSA = body surface area; LVEDVI = indexed left ventricular end diastolic volume; LVESVI = indexed left ventricular end systolic volume; LVEF = left ventricular ejection fraction; LVMI = indexed left ventricular mass; LVSVI = indexed left ventricular stroke volume; BPM = beats per minute.

|  | **Full Cohort**  **(n = 1258)** | **Males**  **(n =578)** | **Females**  **(n =680)** | **P – Value** |
| --- | --- | --- | --- | --- |
| **Age** (years) | 40.6 ± 12.8 (18 – 80) | 40.6 ± 12.4 (19 – 80) | 40.6 ± 13.2 (18 – 75) | 0.73 |
| **Race / Ethnicity:** |  |  |  | 0.91 |
| Caucasian | 950 (75.5%) | 440 (76.1%) | 510 (75.0%) |  |
| South Asian | 155 (12.3%) | 80 (13.8%) | 75 (11.0%) |  |
| African | 84 (6.7%) | 29 (5.0%) | 55 (8.1%) |  |
| Other | 69 (5.5%) | 29 (5.0%) | 40 (5.9%) |  |
| **Systolic BP** (mmHg) | 119 ± 14 | 125 ± 13 | 115 ± 14 | ***<0.001*** |
| **Diastolic BP** (mmHg) | 79 ± 10 | 80 ± 9 | 78 ± 9 | ***<0.001*** |
| **Height** (cm) | 170 ± 9 | 176 ± 7 | 164 ± **7** | ***<0.001*** |
| **Weight** (kg) | 71 ± 13 | 78 ± 12 | 65 ± 11 | ***<0.001*** |
| **BSA** (m^2^ ) | 1.8 ± 0.2 | 2 ± 0.2 | 1.7 ± 0.2 | ***<0.001*** |
| **LVEDVI** (ml/m^2^) | 80 ± 13 (44 – 129) | 85 ± 14 (48 – 129) | 76 ± 11 (44 – 110) | ***<0.001*** |
| **LVESVI** (ml/m^2^) | 28 ± 8 (12 – 66) | 31 ± 8 (12 – 66) | 26 ± 6 (11 – 57) | ***<0.001*** |
| **LVSVI** (ml/m^2^) | 52 ± 8 (32 -88) | 54 ± 9 (33 – 88) | 50 ± 7 (32 – 76) | ***<0.001*** |
| **LVEF** (%) | 65 ± 5 (47 – 81) | 64 ± 5 (48 – 80) | 67 ± 5 (47 – 81) | ***<0.001*** |
| **LVMI** (g/m^2^) | 62 ± 14 (32 – 141) | 71 ± 14 (35 – 141) | 55 ± 10 (32 – 100) | ***<0.001*** |
| **Cardiac Output** (L) | 6.1 ± 1.4 | 6.6 ± 1.5 | 5.6 ± 1.2 | ***<0.001*** |
| **Heart Rate** (BPM) | 65 ± 10 | 63 ± 11 | 66 ± 9.6 | ***<0.001*** |

**Online Table 2:** **Summary of linear regression models for the whole cohort**. R^2^ for LVEDV model: 0.56; R^2^ for LVESV model: 0.42; R^2^ for LVSV: 0.52; R^2^ for LVEF model: 0.14; R^2^ for LVM model: 0.62; R^2^ for Cardiac Output model: 0.33; R^2^ for Heart Rate model: 0.02. BP = blood pressure; BSA = body surface area; LVEDV = left ventricular end diastolic volume; LVESV = left ventricular end systolic volume; LVSV = left ventricular stroke volume; LVEF = left ventricular ejection fraction; LVM = left ventricular mass; Race C = Caucasian; Race AF = African; Race SA = South Asian.

|  |  | **Full Cohort**  **(n = 1258)** | |
| --- | --- | --- | --- |
|  |  | **Standardised β** | ***p*** |
| **LVEDV** | |  |  |
|  | Age | -0.26 | ***<0.001*** |
|  | BSA | 0.46 | ***<0.001*** |
|  | Gender | 0.27 | ***<0.001*** |
|  | Race: C v AF | -0.07 | ***<0.001*** |
|  | Race: C v SA | -0.21 | ***<0.001*** |
|  | Race: C v Other | -0.03 | 0.08 |
|  | Systolic BP | 0.05 | ***0.02*** |
| **LVESV** | |  |  |
|  | Age | -0.26 | ***<0.001*** |
|  | BSA | 0.32 | ***<0.001*** |
|  | Gender | 0.34 | ***<0.001*** |
|  | Race: C v AF | -0.04 | 0.10 |
|  | Race: C v SA | -0.18 | ***<0.001*** |
|  | Race: C v Other | -0.02 | 0.27 |
|  | Systolic BP | -0.05 | 0.07 |
| **LVSV** | |  |  |
|  | Age | -0.21 | ***<0.001*** |
|  | BSA | 0.50 | ***<0.001*** |
|  | Gender | 0.16 | ***<0.001*** |
|  | Race: C v AF | -0.08 | ***<0.001*** |
|  | Race: C v SA | -0.21 | ***<0.001*** |
|  | Race: C v Other | -0.04 | 0.06 |
|  | Systolic BP | 0.12 | ***<0.001*** |
| **LVEF** | |  |  |
|  | Age | 0.19 | ***<0.001*** |
|  | BSA | -0.00 | 0.95 |
|  | Gender | -0.30 | ***<0.001*** |
|  | Race: C v AF | -0.02 | 0.42 |
|  | Race: C v SA | 0.06 | ***0.02*** |
|  | Race: C v Other | 0.01 | 0.65 |
|  | Systolic BP | 0.17 | ***<0.001*** |
| **LVM** | |  |  |
|  | Age | -0.17 | ***<0.001*** |
|  | BSA | 0.38 | ***<0.001*** |
|  | Gender | 0.40 | ***<0.001*** |
|  | Race: C v AF | 0.06 | ***0.001*** |
|  | Race: C v SA | -0.16 | ***<0.001*** |
|  | Race: C v Other | -0.04 | ***0.03*** |
|  | Systolic BP | 0.14 | ***<0.001*** |
| **Cardiac Output** | |  |  |
|  | Age | -0.23 | ***<0.001*** |
|  | BSA | 0.41 | ***<0.001*** |
|  | Gender | 0.04 | 0.23 |
|  | Race: C v AF | -0.08 | ***<0.001*** |
|  | Race: C v SA | -0.15 | ***<0.001*** |
|  | Race: C v Other | -0.03 | **0.19** |
|  | Systolic BP | 0.19 | ***<0.001*** |
| **Heart rate** | |  |  |
|  | Age | -0.06 | 0.06 |
|  | BSA | -0.04 | 0.28 |
|  | Gender | -0.14 | ***<0.001*** |
|  | Race: C v AF | -0.03 | 0.25 |
|  | Race: C v SA | 0.05 | 0.08 |
|  | Race: C v Other | -0.00 | 0.86 |
|  | Systolic BP | 0.13 | ***<0.001*** |
